# Recent Advances of Electrode Materials Applied in an Electrochromic Supercapacitor Device

**DOI:** 10.3390/molecules30010182

**Published:** 2025-01-05

**Authors:** Qingfu Guo, Chao Sun, Yiran Li, Kaoxue Li, Xishi Tai

**Affiliations:** 1 College of Chemistry and Chemical Engineering, Weifang University, Weifang 261061, China; liyr1987@foxmail.com (Y.L.); likaoxue@163.com (K.L.); 2 Shandong Peninsula Engineering Research Center of Comprehensive Brine Utilization, Weifang University of Science and Technology, Weifang 262700, China; kdsunchao@163.com

**Keywords:** electrochromic supercapacitor, electrochromism, energy storage, electrode materials

## Abstract

An electrochromic supercapacitor device (ESD) is an advanced energy storage device that combines the energy storage capability of a supercapacitor with the optical modulation properties of electrochromic materials. The electrode materials used to construct an ESD need to have both rich color variations and energy storage properties. Recent advances in ESDs have focused on the preparation of novel electrochromic supercapacitor electrode materials and improving their energy storage capacity, cycling stability, and electrochromic performance. In this review, the research significance and application value of ESDs are discussed. The device structure and working principle of electrochromic devices and supercapacitors are analyzed in detail. The research progress of inorganic materials, organic materials, and inorganic/organic nanocomposite materials used for the construction of ESDs is discussed. The advantages and disadvantages of various types of materials in ESD applications are summarized. The preparation and application of ESD electrode materials in recent years are reviewed in detail. Importantly, the challenges existing in the current research and recommendations for future perspectives are suggested. This review will provide a useful reference for researchers in the field of ESD electrode material preparation and application.

## 1. Introduction

With the development of social economy, the increase in energy consumption has brought about serious environmental pollution, which seriously threatens human health and environmental safety. In order to achieve sustainable development, a variety of clean and efficient energy sources have been developed, such as solar energy, geothermal energy, wind energy, nuclear energy, etc. [1,2]. However, these indirect energy supplies require an efficient storage system to ensure secure and consistent access to electricity. Supercapacitors and batteries, as the two main electric energy storage systems, have been widely used in different fields from portable electronic devices to smart grids [3,4]. Compared with batteries, a supercapacitor has an impressive energy density, an extended lifespan, rapid charge/discharge capabilities, and almost no maintenance costs [5,6]. At the same time, supercapacitors, as electrochemical capacitors, have the advantages of small size, light weight, adaptability to harsh environmental conditions, and environmental friendliness and play an important role in high specific power and specific energy [7]. They can be used as the power supply for various portable electronic devices and have good application prospects [8,9].

Electrochromism, which causes a change in color by applying an electric current or potential to a material, has a wide range of applications in light regulation and display [10]. In recent years, electrochromism has shown broad application prospects in the fields of intelligent windows, information displays, electronic paper, and electrochromic electronic skin in energy-saving buildings [11,12]. Currently, the design of multifunctional materials is of importance and immediacy to cater to the impending energy storage demands of the future. Specifically, merging energy storage capabilities with electrochromic properties into a singular device holds immense practical significance. Given that both electrochemical energy storage and electrochromic devices rely on the oxidation–reduction processes of electrode materials and share comparable structural designs, the development of electrochromic energy storage devices is viable [13].

The integration of electrochromism with supercapacitors brings together the energy storage capabilities of supercapacitors with the visual energy state indication provided by electrochromic materials. This amalgamation not only enhances the functionality of the device but also offers a novel approach to monitoring the charge state in real time. The electrochromic component in these hybrid devices typically serves as one of the electrodes and undergoes a color change corresponding to the charge and discharge cycles of the supercapacitor [14,15]. In these integrated systems, the electrochromic material is often deposited on a transparent conducting substrate, such as indium tin oxide (ITO) glass, which serves as the current collector [16]. During the charging process, the applied voltage induces redox reactions in the electrochromic material, leading to a change in color. This visual cue indicates the energy level of the supercapacitor, providing direct and intuitive means to assess its state of charge without the need for external measuring devices [17,18]. Recently, lots of electrochromic energy storage devices such as ESD and electrochromic batteries have been reported, and this research field has attracted the attention of researchers [19].

Generally, ESD electrode materials are classified as organic or inorganic materials. Inorganic materials, organic molecules, conducting polymers, and covalent organic frameworks (COFs) have been extensively studied. These types of organic materials are characterized by their rich color palettes, rapid response, and ease of manufacture by solution treatment [20]. However, organic materials exhibit poor thermal and chemical stability compared to inorganic materials, thus limiting their practical application [21]. Inorganic materials have long-term durability, wide operating temperature range, and good chemical stability. However, due to the low electrical conductivity, high volume expansion during cycling, and low ion transport efficiency of inorganic materials, inorganic ESD electrode materials have the disadvantages of slow color response and low energy storage capacity [22]. In this review, the research progress of inorganic materials, organic materials, and inorganic/organic composite materials in electrochromic supercapacitors are discussed, and we explore the research direction and application of electrochromic supercapacitor electrode materials.

## 2. Fundamentals of Electrochromic and Supercapacitor Devices

### 2.1. Supercapacitor Devices

Typically, a supercapacitor consists of two electrodes, electrolytes, and a diaphragm. The performance parameters of supercapacitors are mainly evaluated by several factors, including the specific capacitance, power density, high energy density, voltage window, cycle life, charge and discharge rate, etc. [23]. Electrode materials play a crucial role in the performance of supercapacitors. According to the different energy storage mechanisms, supercapacitors can be divided into two categories, namely electrical double-layer capacitors and pseudocapacitors [24].

The energy storage mechanism of electrical double-layer capacitors is generated by the charge confrontation caused by the directional arrangement of electrons or ions at the electrode/solution interface [25]. For an electrode/solution system, a double electric layer is formed at the interface of an electrically conductive electrode and an ionic conductive electrolyte solution. When the electric field is applied to the two electrodes, the negative and cation in the solution migrate to the positive and negative electrodes, respectively, forming a double electric layer on the electrode surface. After the electric field is withdrawn, the positive and negative charges on the electrode attract the opposite charge ions in the solution so that the double electric layer is stabilized, and a relatively stable potential difference is generated between the positive and negative electrodes [26]. At this time, for a certain electrode, it will generate an equal amount of opposite ionic charge with the charge on the electrode within a certain distance (the dispersion layer) so that it can maintain electric neutrality. When the two poles are connected with the external circuit, the charge on the electrode migrates and generates a current in the external circuit, and the ions in the solution migrate to the solution and are electrically neutral, which is the charge and discharge principle of the electrical double-layer capacitors. Carbon-based materials (carbon nanotubes, activated carbon, etc.) have the characteristics of controllable structure, high conductivity, large surface area, etc. and are generally considered ideal electrode materials for the preparation of electrical double-layer capacitors [27].

The energy storage mechanism of pseudocapacitors is that highly reversible chemical absorption, deactivation, and redox reactions occur in electroactive substances on the electrode surface and near the surface or in the bulk phase, thereby generating capacitance related to the charging potential of the electrode [28]. For pseudocapacitors, the process of charge storage includes not only the storage on the double electric layer but also the redox reaction between the electrolyte ions and the electrode active substances. When ions in the electrolyte (such as H^+^, OH^-^, K^+^, or Li^+^) are diffused from the solution to the electrode/solution interface under the action of an applied electric field, they enter the bulk phase of the active oxide on the surface of the electrode through the redox reaction at the interface, so that a large amount of charge is stored in the electrode. When discharged, the ions entering the oxide are returned to the electrolyte through the reverse reaction of the above redox reaction, and the stored charge is released through the external circuit [28]. In general, pseudocapacitors can store more charge than electrical double-layer capacitors. However, the kinetic process of pseudocapacitors is slower than that of electrical double-layer capacitors. Conducting polymers and transition metal oxides are commonly used electrode materials for pseudocapacitors [29].

### 2.2. Electrochromic Devices

**Figure 1:**
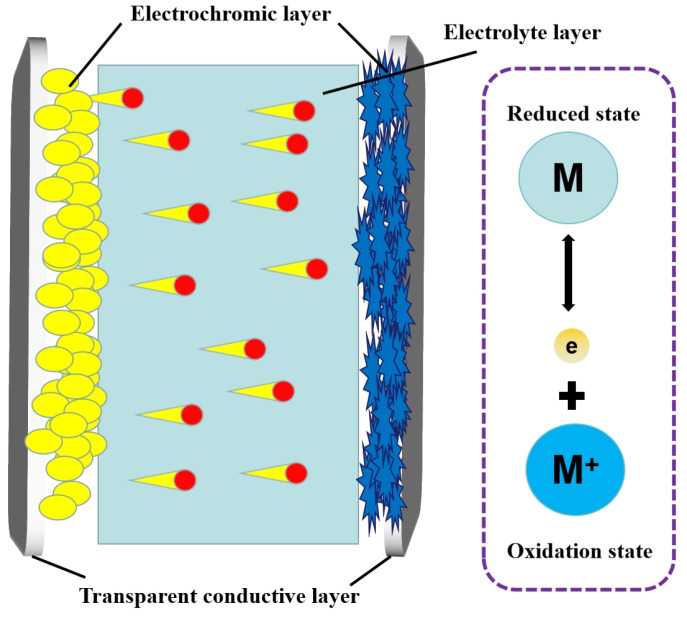
Structure diagram of electrochromic device and the working principle of the electrochromic material.

Electrochromic devices are usually constructed with two transparent conductive layers (electrode layers); sandwiched between them are the electrochromic layer and electrolyte layer Figure 1) [30]. The electrochromic layer is the core layer of the electrochromic device and is also the layer where the color change reaction occurs. When the voltage is applied to the transparent conductive film, the positive and negative ions in the electrolyte film will migrate to the color-changing layer and react with the electrochromic material in the color-changing layer to become colored compounds so that the electrochromic device will show color. When the electrode polarity is opposite, the ion migration direction is opposite, and the electrochromic device is restored to its original state [31]. The electrolyte layer is a channel for ion migration. Under the action of the external electric field, the ions in the electrolyte layer can be migrated, which is an essential part of the electrochromic device to realize the color-changing function [32]. The working principle of the electrochromic material with the chemical reaction can be expressed as “M (reduced state) → M^+^ (Oxidation state)+e”, which works in the same way as the energy storage mechanism of electrode materials for pseudocapacitors (Figure 1) [33]. The electrochromic material displays different colors in the oxidized and reduced states respectively. The combination of UV-Vis spectroscopy and electrochemistry is an important means of studying the electrochromic properties of conductive polymer films. For an ideal electrochromic material, it should have both a large transmittance and a small electrical variation. Contrast, coloring efficiency, response time of color development, and optical memory are important parameters for evaluating an electrochromic material [34]. At present, electrochromic devices have shown broad application prospects in the fields of smart windows, self-powered batteries, and solar panels because of their unique optical properties [35].

Although electrochromic technology has made some progress, it still faces some challenges. The response speed of electrochromic devices is a major limiting factor in their practical applications [36]. In order to improve the response speed, the adjustment strategies can be studied from two aspects: electrochromic material and electrolyte. For example, by using electrolyte modulation strategies such as polyvalent ions, proton relay transport and mixed ion synergy, as well as using composite electrode and porous electrochromic materials, the migration of transferred ions can be accelerated, thus improving the response speed of the device. Another challenge for the application of electrochromic devices is the large area preparation [37]. At present, the preparation method of large-area electrochromic devices is the traditional screen printing method, but there are some problems in the assembly process, such as the electrolyte slurry formula viscosity being too small, which will lead to poor shape retention during the assembly process, and the viscosity being too large, which will make the slurry screen penetration poor and unable to print the corresponding pattern [38,39]. In order to solve this problem, a new method to print the electrolyte layer can be proposed by mixing the electrolyte slurry with diluent so that the viscosity of the electrolyte diluent meets the requirements of screen printing and removing the diluent after printing, improving the viscosity of the printing layer so that the printing layer has a certain rigidity and a certain wettability. It can maintain its shape to inhibit elongation during the bonding process and can ensure that the uncured electrolyte layer can be in close contact with other functional layers [40,41]. Although electrochromic devices still face some challenges in practical applications, these problems are expected to be gradually solved through continuous research and technical improvement, thus promoting the further development and application of electrochromic technology.

## 3. Inorganic Materials

### 3.1. Transition Metal Oxides

Researchers have put a lot of effort into promoting the use of various transition metal oxides for supercapacitors. Their choice can be found in the unique characteristics of metal oxides, such as ideal capacitive performance, low cost, and environmental friendliness [42]. Their charge storage mechanism follows pseudocapacitance behavior. At the same time, due to its reversible redox reaction, high energy density, excellent specific capacitance, and metal ions in different valence states of the material to show different color changes, inorganic metal oxides are usually used as the representative electrode materials of ESD [43].

#### 3.1.1. Tungsten Oxide

**Figure 2:**
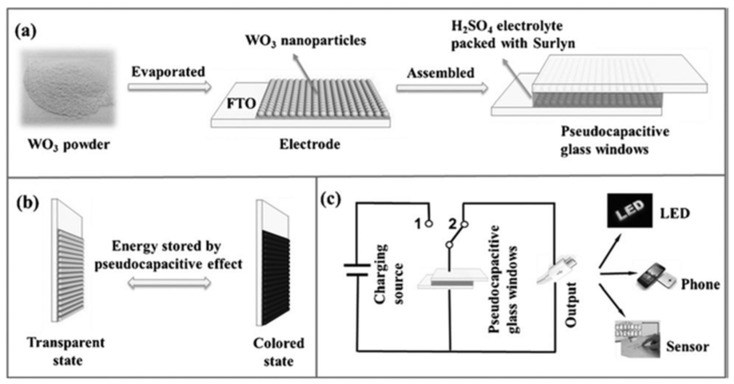
(**a**) Preparation of the WO_3_ and the structure of the device; (**b**) Electrochromism of the WO_3_ electrode; (**c**) Working principle of the energy storage intelligent window [48]. Copyright 2014, Wiley Online Library.

Tungsten oxide (WO_3_) stands as a pioneering inorganic compound discovered to possess electrochromic characteristics, along with excellent electrochemical properties; it has garnered considerable research attention within the realm of ESD [44]. Xie’s group has demonstrated the efficacy of Mo-doped WO_3_ amorphous films in energy storage applications [45]. Nonetheless, enhancements are required in the diffusion rate, surface capacitance, and light modulation capabilities of WO_3_ layers to better align them with the demands of energy conservation and optical utilization. Numerous methodologies for synthesis and diverse deposition parameters have been employed to refine the characteristics of WO_3_ coatings. Tu’s group designed a multifunctional thin film with both supercapacitor and electrochromic properties by using molybdenum-doped WO_3_ nanowire arrays [46]. In the constant current charging and discharging process of molybdenum-doped WO_3_ nanowire arrays, the thin films show obvious color change and transmittance modulation. When the potential is increased from −0.7 V to 0.4 V, the specific capacitance of the material reaches 55.89 F g^−1^. At 750 nm, the transmittance increases from 20% to 75%, and the color changes from dark blue to transparent. The coloring efficiency of the molybdenum-doped WO_3_ is 123.5 cm^2^ C^−1^ (the electrochromic and specific capacitance parameters of some reported ESD electrode materials are illustrated in Table 1). These results suggest that WO_3_ nanomaterials have the potential to integrate energy storage and electrochromism into a single device. Malik’s group reported the surface modification of γ-WO_3_ films deposited by room temperature sputtering, which enhanced the reversible redox properties and optical modulation [47]. Mai’s group successfully prepared ultra-thin WO_3_ films using a simple thermal evaporation WO_3_ powder on the FTO. The ESD constructed by the WO_3_-based electrode demonstrated remarkable electrochemical properties, including a max specific capacitance of 639.8 F g^−1^. Upon the application of a 0.6 V potential, the hue of the WO_3_ layer underwent a transformation from transparent to dark blue, with a dramatic decrease in the light transmission at 633 nm, plummeting from an initial 91.3% to a mere 15.1%. The energy storage intelligent window with a large energy storage capacity based on WO_3_ has also been successfully prepared (Figure 2) [48]. This transformation paves the way for the integration of intelligent electrochromic windows into portable electronic devices.

**Figure 3:**
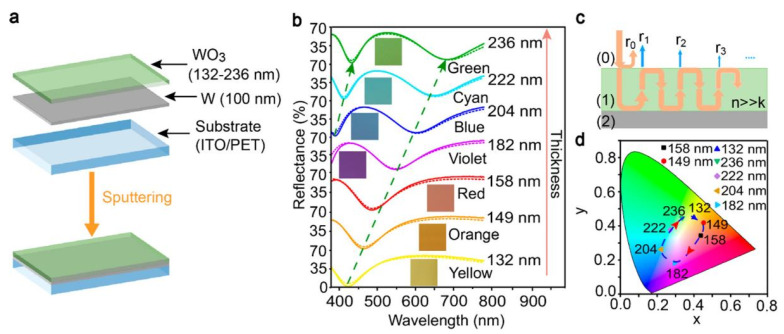
(**a**) Schematic diagram of the structure for the F-P cavity-type ESD electrode; (**b**) Simulated and measured reflection spectra and the optical images of the electrodes with different thicknesses of WO_3_; (**c**) Schematic diagrams of light wave reflection processes of the ESD electrodes; (**d**) CIE 1931 color coordinates of the F-P cavity-type ESD electrodes with different thicknesses of the WO_3_ layer [49]. Copyright 2020, American Chemical Society.

Although tungsten oxide has been widely used in ESD, it is difficult to achieve acceptable visual aesthetics because this material can only present a color palette from transparent to blue. Zhao’s group achieved a breakthrough by documenting the pioneering Fabry Perot cavity ESD that exclusively utilizes WO_3_. This innovative device is capable of showcasing an array of vivid hues and mesmerizing designs that correspond to its charging and discharging cycles [49]. At full charge, these units are capable of serving as an energy source for additional ultracapacitors, adopting distinctive configurations like the shape of dolphins. As depicted in Figure 3, the WO_3_ electrode boasts a coloration efficiency of 140 cm^−2^ C^−1^ and an impressive areal capacitance of 23.4 mF cm^−2^. This groundbreaking finding paves the way for the development of advanced WO_3_-based smart energy storage systems featuring multicolored display capabilities.

#### 3.1.2. Nickel Oxide

Nickel oxide (NiO) can also be used to develop high-performance ESD because of its high transmittance modulation and high cycle stability [50]. Deb’s group pioneeringly crafted a robust nanocomposite ESD with a solid-state structure in 2005 [51]. The composite features active electrochemical elements in the form of polycrystalline NiO nanoparticles, encircled by an amorphous Ta_2_O_5_ proton electrolyte. This achievement paves the way for the development of ESD and extends the horizon for the exploration of capacitors with diverse functionalities. Lee’s group prepared NiO nanoparticle films using the hydrothermal method and used them as electrodes for ESD. At the current density of 1 A g^−1^, the capacitance value of NiO nanoparticle is 1386 F g^−1^, the transmittance modulation at 550 nm is 63.6%, and the coloring efficiency is 42.8 cm^2^ C^−1^ [52]. Mai’s group prepared NiO nanosheet-based electrodes on FTO glass using a simple hydrothermal method [53]. The unique structure of the porous nanosheet, coupled with its robust bonding to the FTO, endows the NiO electrode with superior functionality in both energy storage systems and electrochromic applications. When utilized as a component in a supercapacitor, it boasts an impressive areal capacitance (74.8 mF cm^−2^) and remarkable cyclic stability, withstanding up to 5000 charge–discharge cycles at a scan rate of 10 mV s^−1^. At a wavelength of 632.8 nm, the transmittance modulation rate is as high as 40%, and the color rendering efficiency is as high as 63.2 cm^−2^ C^−1^. High transparency and high conductivity are the requirements for high-rate energy storage and the rapid discoloration of electrode materials.

**Table 1:** The electrochromic and specific capacitance parameters of some reported ESD electrode materials.

Electrode Material	ΔT%	Coloring Efficiency	Specific Capacitance	References
Molybdenun-doped WO_3_	56.7%	123.5 cm^2^ C^−1^	55.89 F g^−1^	[46]
WO_3_ polycrystalline	76.2%	54.8 cm^2^ C^−1^	639.8 F g^−1^	[48]
WO_3_	-	140 cm^2^ C^−1^	68.4 mF cm^−2^	[49]
NiO nanoparticle	63.6%	42.8 cm^2^ C^−1^	1386 F g^−1^	[52]
NiO nanosheet	40%	63.2 cm^2^ C^−1^	74.8 mF cm^−2^	[53]
NiO/Ag/NiO	70%	76.6 cm^2^ C^−1^	364 F g^−1^	[54]
Silver nanowire/NiO	15%	51.9 cm^2^/C	3.47 mF cm^−2^	[55]
PANI nanowire arrays	60%	-	17 mF cm^−2^	[56]
PProDOT-Me_2_	-	-	55 F g^−1^	[57]
PDThOB	-	-	112.4 F g^−1^	[58]
Poly(3,4-dibromothiophene)	43%	357 cm^2^ C^−1^	229.6 F g^−1^	[59]
PCDB-EDOT	25%	158 cm^2^ C^−1^	4.65 mF cm^−2^	[60]
PThQ-Ph	80%	300 cm^2^ C^−1^	1.58 mF cm^−2^	[61]
PC10QA-2EDOT	40%	498 cm^−2^ C^−1^	322 F cm^−3^	[62]
PANI/WO_3_ nanocomposite	35.3%	98.4 cm^−2^ C^−1^	0.025 F cm^−2^	[63]
PANI/MoO_3−x_ composite	33%	-	606 F g^−1^	[64]
PICA/TiO_2_ composite	36%	124 cm^2^/C	23.34 mF cm^−2^	[65]
P5ICA/WO_3_ composite	-	-	30.2 mF cm^−2^	[66]
PB/P5ICA composite	60.1%	262 cm^2^/C	98.12 mF cm^−2^	[67]

**Figure 4:**
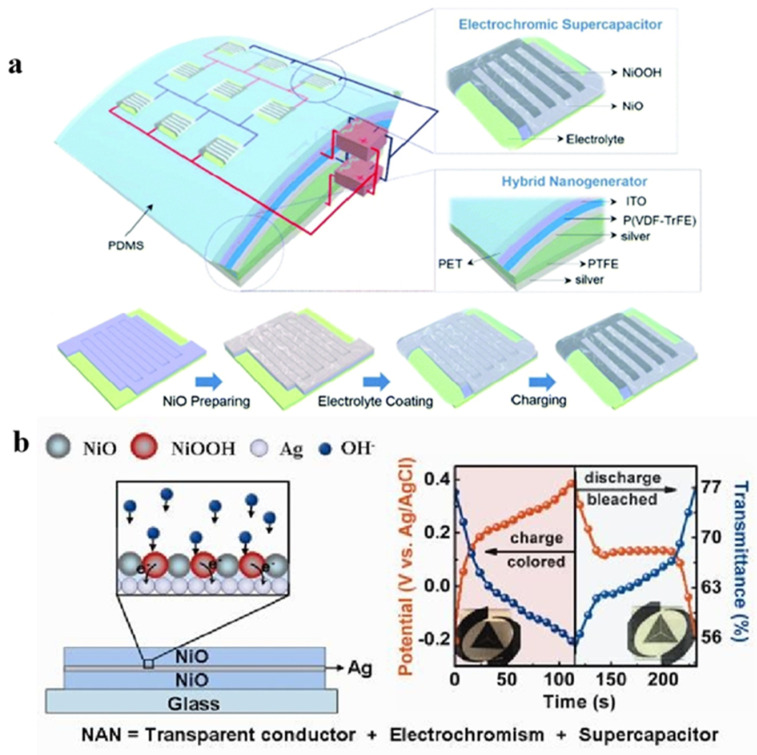
(**a**) Schematic illustration of the flexible and smart self-charging power package [54]. Copyright 2018, Wiley Online Library. (**b**) The structure of transparent ITO-free NiO-Ag-NiO electrode and in situ transmittance and chronocoulometry switching curves with color changes [55]. Copyright 2017, Royal Society of Chemistry.

For the first time, Wang’s group crafted a flexible and smart self-charging power package, merging triboluminescent/piezoelectric nanogenerators with ESD into a unified device. For the functional electrode material, ESD employed silver nanowire/NiO (AgNW/NiO) combinations (Figure 4) [54]. The electrochemical analyses revealed that this system boasted impressive capacitance levels of 3.47 mF cm^−2^ (17.4 F g^−1^) and robust cycling characteristics (80.7% after 10000 cycles). In the process of charging and discharging, the AgNW/NiO electrode undergoes a visible transformation from black to transparent due to the reversible oxidation–reduction process of Ni^2+^/Ni^3+^, demonstrating a coloration efficiency of 51.9 cm^−2^ C^−1^ at the 550 nm wavelength. In the process of self-charging, the module’s charge status can be monitored via the color change, thus enabling the creation of a self-charging power package system. Liu’s group successfully prepared NiO/Ag/NiO (NAN) three-layer stacked films on ITO using a simple electron beam method (Figure 4) [55]. The NAN film has an average transmittance of 70% in the visible region, which is comparable to that of ITO/NiO films (average 77.4%). NAN also has a higher magnification capacity; at 0.8 mA cm^−2^, the specific capacitance retains 62.2% of the initial capacitance at 0.05 mA cm^−2^. After 800 cycles, 89.3% of the initial capacitance value is retained. This lays a foundation for further study of the high-conductivity and transmittance electrode materials.

### 3.2. Inorganic Nonmetallic Oxide

Prussian blue (PB), or ferricyanide, is widely used as a pigment in coatings, paints, and printing ink formulations. Among the highly recognized electroactive inorganic substances, PB stands out for its extensive investigation across various scientific domains including electrochromic behavior, electrochemical catalysis, sensors, and energy conservation systems [68,69]. Characterized as a classic coordination compound, this transition metal hexacyanide complex boasts a cubic crystalline system and an open frame structure formed by alternating Fe^2+^ and Fe^3+^ centers that are bridged by cyanide ions in the Fe^2+^-C triple bond [70,71].

In general, PB is used as an electrode material because it can increase the electrode/electrolyte contact area and improve the electrochemical reaction rate. Lou’s group developed a self-powered electrochromic smart window based on Prussian blue, which can be used as a self-rechargeable battery [72]. They reduced Prussian blue to Prussian white (colorless) with aluminum in a potassium chloride electrolyte, achieving self-bleaching of the energy storage device. At the same time, the device can be self-charged (turning blue again) by simply disconnecting the aluminum electrode and the Prussian blue electrode, which is achieved by the spontaneous oxidation of Prussian white to Prussian blue by dissolved oxygen in an aqueous solution.

**Figure 5:**
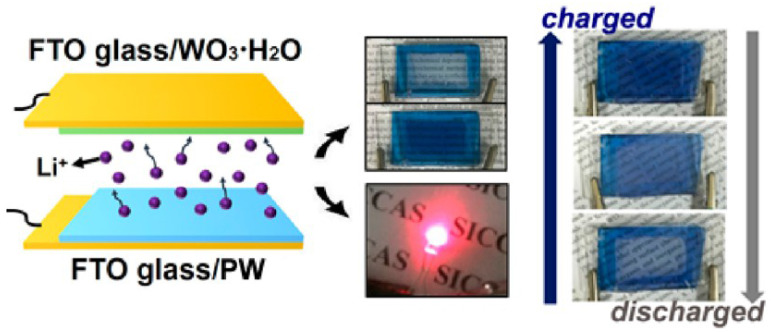
Structure diagram of electrochromic energy storage device based on WO_3_·H_2_O nanosheets and PW film and schematic diagram of charge and discharge [73]. Copyright 2017, American Chemical Society.

Prussian blue appears white in its reducing state, also known as Prussian white (PW). Li’s group developed a novel electrochromic energy storage device composed by WO_3_·H_2_O nanosheets and PW film [73]. This device has a wide optical modulation capability, ultra-fast response time, excellent coloring efficiency and long cycle life. In addition, an enlarged electrochromic energy storage device was built as a prototype to visually monitor the stored energy level through color changes (Figure 5).

## 4. Organic Materials

### 4.1. Organic Conducting Polymer

Organic conducting polymers (CPs) are a class of polymers that can be used in electrochromic devices and supercapacitors due to their good electrochemical activity [74,75]. When used in electrochromic devices, CPs have the advantages of multi-color variation, high optical modulation, fast color response, and high coloring efficiency [76]. In terms of supercapacitors, the electrochemical doping/de-doping process of CPs can obtain high specific capacitance, fast redox kinetics, and good conductivity, which makes CPs excellent electrode materials for supercapacitors [77]. In recent years, the application of conducting polymers in electrochromic supercapacitors is mainly concentrated in polyaniline (PANI), polythiophene (PTh), polyindole (PIn), and their derivatives.

#### 4.1.1. Polyaniline

Polyaniline is a conducting polymer widely used in electrochromes and supercapacitors. It has the advantages of high energy storage capacity, high redox reversibility, stable doping state, low cost, and simple preparation process [78]. In general, at voltages ranging from −0.2 V to 1 V, in H^+^-based electrolytes, polyaniline can exhibit four stable electrochromic redox states: yellow, green, blue, and purple. Wei’s group successfully crafted an innovative, adaptable energy storage smart (ESS) window utilizing PANI nanowire arrays [56]. This ESS window shows a surface capacitance of 0.017 F cm^−2^. When subjected to a sweep rate of 5 mV s^−1^, it maintains a capacitance retention of 90% even after 1000 charge–discharge cycles. Serving as an electrode material for electrochromic applications, it achieves a light transmittance of 30% at 500 nm. By integrating ESS windows with photovoltaic cells, novel intelligent devices can be created. Under intense sunlight, the ESS window can transform solar radiation into electrical energy for self-charging.

**Figure 6:**
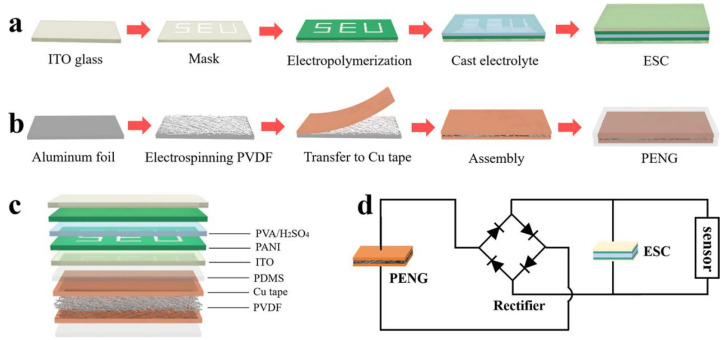
(**a**) Fabrication process of the ESD; (**b**) Preparation process of the piezoelectric nanogenerators; (**c**) Schematic depiction of the self-powered patterned ESD; (**d**) Equivalent circuit of the ESD [79]. Copyright 2018, American Chemical Society.

Gu’s group designed a portable, piezoelectrically-activated, self-sustaining patterned ESD utilizing a PANI-based electrode material (Figure 6) [79]. The obtained ESD has an area-specific capacity of 22.60 mF cm^−2^ under the current density of 0.1 mA cm^−2^. After 1000 cycles, under the current density of 0.3 mA cm^−2^, the specific capacitance remains at 60.34% of the initial capacitance. Using the PANI electrodes assembled a symmetrical ESD when a potential of −1.0–1.0 V is applied, the positive electrode of the polyaniline shows a yellow-green–light green–green-blue–dark blue color change, while the negative electrode shows the opposite color change.

**Figure 7:**
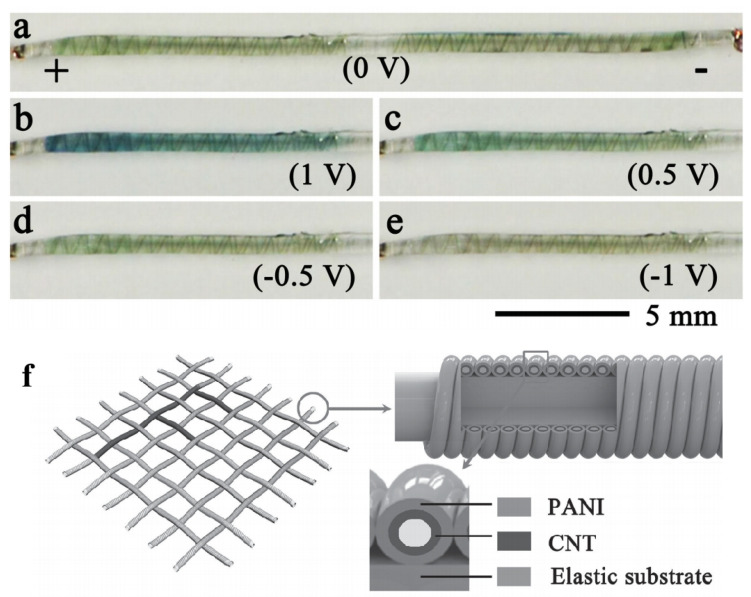
(**a**–**e**) Chromatic transitions during the charge–discharge process under different potentials. (**f**) Schematic illustration of the structure of the fiber-shaped electrochromic supercapacitor [80]. Copyright 2014, Wiley Online Library.

In contrast to the prevalent ESDs that feature flat configurations, ESDs in fibrous forms have scarcely been documented. Peng’s group succeeded in fabricating a fibrous ESD by employing an electrodeposition method to coat PANI onto aligned carbon nanotube sheets (Figure 7) [80]. The device has high energy storage performance, and the specific capacitance can reach 215.6 F g^−1^ under the scan rate of 10 mV s^−1^. The changes in green and blue colors during charge and discharge indicated that the electrochromic and capacitive properties of polyaniline were caused by electrochemical doping/de-doping. The ESD based on PANI can reach 255.5 F g^−1^ (0.1890 mF cm^−2^), the energy density is 12.75Wh kg^−1^, and the power density is 1494 W kg^−1^. ESD can retain a 93.8% capacitance value after 1000 bending and a 97.9% capacitance value after 100 stretching. The textile-based supercapacitor undergoes a visible transformation, changing from blue to green and finally to a light yellow throughout the charge and discharge cycles. These color changes are capable of being cycled over 3000. This phenomenon offers critical insights for the integration of conducting materials and the structuring of electrochemical cell systems in future studies. The capabilities of PANI have been showcased in the realm of advanced smart energy systems, which integrate both electrochromic light control and electrochemical energy retention functionalities. A significant issue that researchers need to address is enhancing the structural integrity of the PANI coating, as it undergoes cyclic anion insertion and removal during the electrochemical oxidation–reduction reactions.

#### 4.1.2. Polythiophene

**Figure 8:**
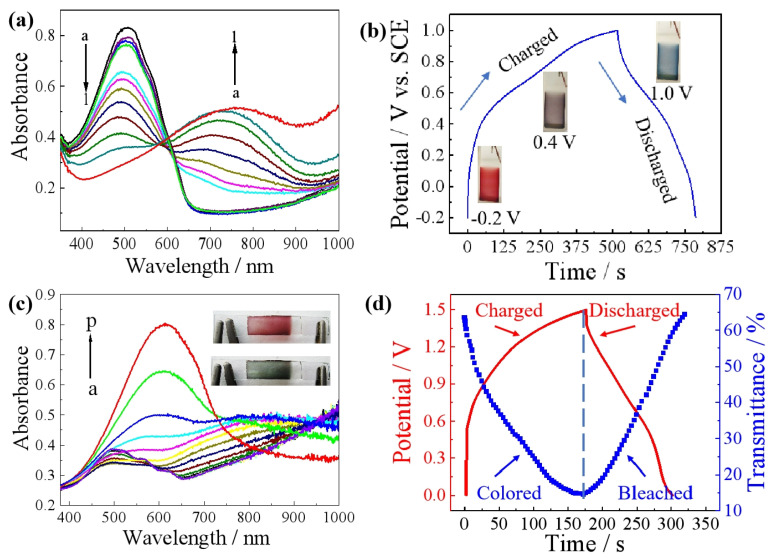
(**a**) Spectroelectrochemistry of the PDBrTh; (**b**) Color change of PDBrTh film during charging and discharging process; (**c**) Spectroelectrochemistry of the ESD based on PDBrTh; (**d**) The relationship between transmittance curve and GCD curve of the ESD [59]. Copyright 2023, Elsevier.

Polythiophene and its derivatives are also used in ESD due to their good electrochromic properties, electrochemical activity, and cycle stability. Poly(2,2-dimethyl-3,4-propyl-dioxthiophene) (PProDOT-Me_2_), an important polythiophene derivative, has multi-color electrochromic properties and good energy storage performance [57,81]. PProDOT-Me_2_ exhibits a dark blue hue under cathode coloration and transitions to a transparent state under anode coloring. Constant current charge–discharge tests revealed that the polymer exhibited good energy storage performance, with a specific capacitance of 55 F g^−1^ at a current density of 0.5 mA cm^−2^. Another novel polythiophene derivative (PDThOB) with good electrochromic and supercapacitor properties was synthesized in a LiClO_4_/propylene carbonate electrolyte by Yuksel’s group [58]. The results show that the polymer exhibits a variety of color variations (fuchsia at 0.2 V and black at 1.3 V) and has a specific capacitance of 112.4 F g^−1^ at a current density of 1.0 A g^−1^. Using boron trifluoride diethyl etherate, our group synthesized poly(3,4-dibromothiophene), a material capable of reversible color shifts between red and blue. The specific capacitance value can reach 229.6 F g^−1^ under the current density of 1 A g^−1^. The constructed all-polythiophenes ESD by the prepared poly(3,4-dibromothiophene) and poly(3,4-ethylenedioxythiophene) also showed good electrochromic, energy storage properties, and robust charge–discharge stability (Figure 8) [59]. A solution-processable thiophenol [3,2-b] thiophenyl donor–acceptor polymer (PBOTT-BTD) was synthesized, and an asymmetric ESC was assembled by Meng’s group [82]. The resulting device has high operating voltage, power density, and good cycling stability and can show color change during charging and discharging. In a related study, Zhang’s group designed and synthesized a novel twisted donor–acceptor–donor (D–A–D) poly(chalcogena-diazolobenzotriazole-3,4-ethylenedioxythiophene) (PCDB-EDOT) with an area-specific capacitance up to 4.65 mF cm^−2^. The assembled ESD can light a single yellow LED (1.8 V, 0.04 W) for more than 60 s and has good cycle stability [60]. Liu’s group synthesized three D–A–D-type monomers based on thiophene-substituted quinoxaline derivatives (ThQ-Ph, ThQ-PhOMe and ThQ-Th), and the corresponding conducting polymer films were prepared by electropolymerization [61]. The polymer film exhibited tunable polychromatic electrochromic in visible light and near-infrared with an optical contrast of more than 80% at 1600 nm, a short response time (less than 1.5 s), and a coloring efficiency as high as 300 cm^2^ C^−1^. The ESD exhibited a specific capacitance of 0.81–1.58 mF cm^−2^ at the current density of 0.01 mA cm^−2^. A range of quinacridone (QA) compounds featuring varied donating groups (C10QA-2T, C10QA-2EDOT, C10QA-2DT) were meticulously designed and prepared by Zhang’s group [62]. Subsequent to this, the corresponding polymers were synthesized through the process of electrochemical polymerization. Compared with pC10QA-2T and pC10QA-2DT, pC10QA-2EDOT films have higher optical pair ratio (over 40% in the visible light region), coloring efficiency (498 cm^−2^ C^−1^), specific capacitance (322 F cm^−3^), and cycle stability. An ESD based on pC10QA-2EDOT showed good stability after 50000 cycles, and its initial optical contrast is almost no attenuation.

### 4.2. Viologen Small Molecules

**Figure 9:**
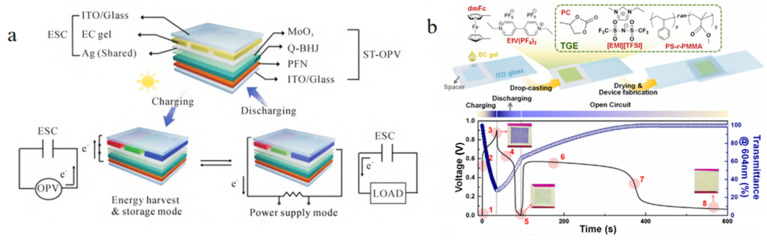
(**a**) ST-OPVs monolithically integrated with ESC based on viologen derivatives [84]. Copyright 2020, Wiley Online Library. (**b**) Schematic of the fabrication process of ternary gel electrolyte-based ESD, the GCD curves, and the corresponding transmittance profile [85]. Copyright 2021, Elsevier.

Viologen is one of the representatives of oxidation-reduced electrochromic materials. Feng’s group reported a stimulation-responsive miniature supercapacitor (SR-MSC) with ultra-high energy density and ultra-short switching time by using methyl viologen as the color-changing material and graphene nanosheet/V_2_O_5_ hybrid nanopaper as the electrode [83]. By integrating the translucent four-element composite organic photovoltaic cell (STQ-OPVs) with the ESC based on the viologen derivative gel, a pattern-based translucent energy storage photovoltaic device that can run all day was prepared by Ko’s group, as shown in Figure 9a [84]. The absorption tunability of the four-element organic complex and the low power consumption of ESD facilitate the device to achieve color change, energy collection, and storage under various lighting conditions. In addition, the effect of ternary gel electrolytes containing small organic molecules on the performance of ESC was studied by Moon’s group (Figure 9b) [85]. The diffusion control mechanism of integrated ESD based on a single color-changing gel layer was elucidated. Electrochromic devices can change their optical transmittance at an external power source but cannot spontaneously modulate the optical flow in real time as the intensity of the surrounding light changes. In order to solve this problem, an ESD powered by perovskite solar cells based on viologen derivative gel is constructed. This device can achieve rapid adjustment of transmittance in the visible-infrared region, so as to adjust the light energy flow in real time according to the surrounding light intensity.

## 5. Inorganic/Organic Composite Materials

In the application of ESD, inorganic materials possess high theoretical specific capacitance and superior cyclic stability, albeit with a limited color transformation. Conversely, organic materials exhibit distinct benefits in electrochromic properties, including multicolor redox state, enhanced light adjustment capabilities, high color efficiency, and swift transition dynamics, along with notable electrical conductivity and flexibility, but the long-term cycle stability is poor, which is not conducive to practical applications. Organic/inorganic composite materials can take advantage of the synergistic advantages of inorganic materials and organic materials to improve the overall electrochemical properties, electrochromic properties, and cyclic stability of the materials. At present, inorganic-organic composite materials have been widely studied and applied in ESD.

### 5.1. Transition Metal Oxides/Conducting Polymer Composite Materials

In recent years, researchers have prepared a variety of ESDs based on transition metal oxides/conducting polymer composite materials (such as TiO_2_/PANI [86], WO_3_/PEDOT: PSS [87], W_18_O_49_/PANI [88], SiO_2_/PANI [89]). Guo’s group successfully prepared PANI/WO_3_ nanocomposite on ITO electrode through three steps of spin coating, annealing, and electropolymerization [63]. The transmittance modulation rate of PANI/WO_3_ composite is 35.3%, and the area-specific capacitance is 0.025 F cm^−2^, which is similar to PANI, but obviously better than pure WO_3_. The composite material has a higher color efficiency (98.4 cm^−2^ C^−1^) and good cycling stability (current density remains unchanged after 1000 cycles) compared to the pure single material because the synergistic effect between the PANI and WO_3_ particles plays an important role. In addition, Qin’s group prepared a PANI/MoO_3−x_ core-shell composite by in situ chemical oxidation polymerization, and the corresponding ESD was assembled [64]. The synergistic effect of PANI nanorods and MoO_3−x_ nanoribbons promoted the charge transport of PANI and reduced the volume change of PANI, thus improving the rate performance and cycle stability of PANI. In the composite material, the shell of PANI nanorods gives the complex electrode a higher specific capacitance (606 F g^−1^ at 1 A g^−1^ current density, 424 F g^−1^ at 20 A g^−1^ current density) and a higher optical ratio (33%). Using a MoO_3-x_ nanoribbon as the core gives the composite electrode a short color-changing response time, good rate performance, and cycle stability (capacitance retention of 80.1% after 2000 cycles). Compared with a single PANI electrode, the optimized PANI/MoO_3−x_ has higher specific capacitance, optical contrast, magnification performance, and cycle stability. The flexible symmetric ESD assembled by PANI/MoO_3−x_ has a higher energy density and still shows good flexibility after 100 180° bending cycles.

**Figure 10:**
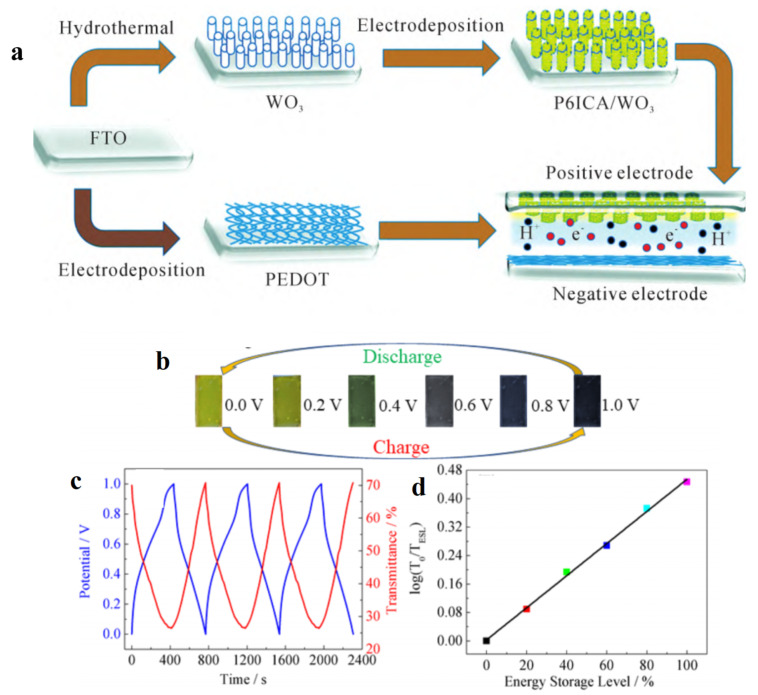
(**a**) Schematic diagrams of the preparation procedure of P6ICA/WO_3_/PEDOT ESD [90]. Copyright 2020, Royal Society of Chemistry. (**b**) Color photos of the ESD under different voltages. (**c**) The relationship between transmittance change and GCD curve. (**d**) The linear relationship between optical densities and ESL of the ESD [66]. Copyright 2020, American Chemical Society.

Our group has also done some research on the preparation of transition metal oxides/polyindole nanocomposites and their application in ESD. For example, poly(indole-6-carboxylic acid) (P6ICA)/TiO_2_ difunctional porous composites were prepared utilizing TiO_2_ nanorods as scaffolds [65]. Due to the co-action of P6ICA and TiO_2_, the prepared P6ICA/TiO_2_ composites exhibit good electrochemical properties, high specific capacitance (23.34 mF cm^−2^), and excellent constant current charge–discharge stability. A non-symmetric ESD was constructed using P6ICA/TiO_2_ nanocomposites as the positive pole and PEDOT as the negative pole. The ESD has good cycle stability and high specific capacitance and can be switched from light green to dark blue. P6ICA/WO_3_ core-shell nanorod composites were also prepared by hydrothermal synthesis and electrodeposition, as shown in Figure 10a [90]. WO_3_ nanorods have a large specific surface area, which is conducive to improving the charge transfer rate. After the electrodeposition of P6ICA on WO_3_ nanorods, the P6ICA/WO_3_ core-shell composite showed higher conductivity and electrochemical stability, and the ion diffusion and electron transfer rates were also improved during the redox process. The nano-composite material can show multiple color transformations (dark green, yellow, and yellow-green), and its specific capacitance is up to 33.8 mF cm^−2^. The ESD assembled by P6ICA/WO_3_ has an optical contrast ratio of 62% at 625 nm, a coloring efficiency of 763cm^2^ C^−1^, and a specific capacitance of 13.69 mF cm^−2^. In order to accurately and quantitatively derive the energy storage percentage of electrochromic supercapacitors, our group successfully prepared poly(indole-5-carboxylic acid) (P5ICA)/WO_3_ nanocomposites with good energy storage and electrochromic properties by using P5ICA composite with WO_3_ and constructed intelligent ESD [66]. The optical density and energy are established based on the real-time transmittance measurement results of intelligent supercapacitors. Based on the linear relationship of the energy storage percentage, the energy storage percentage of intelligent supercapacitors can be accurately and quantitatively deduced by the real-time measurement of light transmittance and the conversion of optical density (Figure 10b–d). The quantitative monitoring of the energy storage state of supercapacitors can be realized.

Current research on ESD electrode materials mainly focuses on inorganic/organic composites. By integrating the advantages of the inorganic and organic material and using the theory of double electric layer and Faraday pseudocapacitance to store charge, it is expected to obtain ESD with higher capacitance and higher energy density. Therefore, the key to the application of inorganic/organic composites in ESD is to improve the structure of composites, optimize the preparation process reasonably, and pay attention to the effect of structural changes on their electrochemical properties.

### 5.2. Other Inorganic/Organic-Based Composite Materials

**Figure 11:**
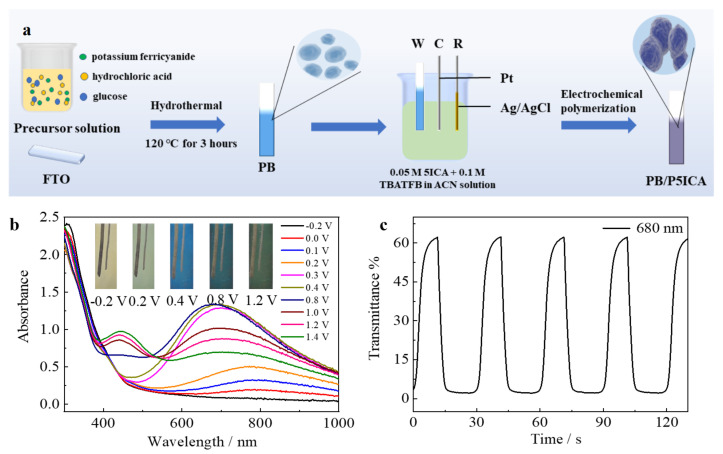
(**a**) The synthesis procedure of PB/P5ICA; (**b**) The color change under different potentials of PB/P5ICA; (**c**) The transmittance curve of the PB/P5ICA [67]. Copyright 2022, Elsevier.

In other inorganic/organic-based composite materials, PB is often used to prepare high-performance electrochromic supercapacitor electrode materials by combining with other organic materials due to its good energy storage and electrochromic properties. Wang et al. designed and synthesized a self-doping network polysiloxane with oligoaniline and sulfanilamide (TASA) through an electrochemically assisted hydrolysis crosslinking reaction [91]. PB is then deposited directly on the porous and interpenetrating TASA membrane to generate an electroactive inorganic–organic double-layer composite (TASA/PB). The prepared TASA/PB electrodes show large changes in transmittance, uniform color changes (transmitted light yellow, light green, blue-green, blue, and dark blue), and good switching durability. In addition, TASA/PB electrodes also have high specific capacitance and charge storage visualization, demonstrating excellent supercapacitor performance. Our group successfully prepared PB/P5ICA nanocomposites with multiple-layer nanosphere structures, which can be reversibly changed among light yellow, light green, blue, and yellow-green, as shown in Figure 11. The prepared PB/P5ICA nanocomposites also have good energy storage performance and cycle stability. A symmetric ESD is constructed based on the PB/P5ICA. The ESD’s energy storage state can be visually monitored through the color change of the electrode material [67].

The PEDOT/titanium carbide (Ti_3_C_2_T_x_) micro-ESDs were installed by Gogotsi’s group, which opened a new way for the development of MXene-conducting polymer heterostructures for electrochromic energy storage devices [92]. Lin’s group synthesized a novel solution-processable composite material (MWCNT-PBDTC), which was well dispersed in common organic solvents and easy to form films [93]. With prepared MWCNT-PBDTC film as an electrode and LiClO_4_/PC-PMMA as an electrolyte, the constructed ESD had a high energy density (174.7 Wh kg^−1^), power density (4.8 kW kg^−1^) and wide operating voltage window (4.8 V). The specific capacitance of the device can remain above 96% of the initial value after 5000 charge and discharge cycles.

## 6. Current Research Challenges

At present, electrochromic supercapacitors have become one of the hot spots in the research field of multifunctional supercapacitors. So far, some progress has been made in the research on the electrode material type, device structure, and application of high-performance electrochromic supercapacitors, but there are still some challenges in the current electrochromic supercapacitors.

Firstly, the capacitance value is limited. When the device is working, it needs high coloring efficiency and fast response time, which requires low charge density to adjust the light contrast of the material. In order to achieve a good energy storage effect, supercapacitors require high charge storage capacity, which leads to a contradiction between energy storage and discoloration. At present, the research of electrochromic supercapacitors mainly uses the electrochromic properties of electrode materials to realize intelligence and pays less attention to the energy storage performance of devices.

Secondly, intelligence is not really realized. At present, the color change of the electrode material prepared is relatively simple, mostly consisting of two colors, and the intelligent visualization is not obvious; no clear quantitative relationship has been established between the color change parameters (such as transmittance, optical density, etc.) of the device and the energy storage level. Therefore, it is very important for the development of electrochromic supercapacitors to design and synthesize new multi-chromic electrode materials with good electrochromic and energy storage performance and realize the quantitative monitoring of the device energy storage levels.

Thirdly, the cycle stability of electrode materials is limited. Recent research has explored organic/inorganic hybrid materials, which have shown improved stability and performance. However, addressing this issue also necessitates the development of novel materials with enhanced durability, such as novel metal oxides, metal-organic frameworks, and perovskites. These materials may offer superior performance and stability.

## 7. Future Perspectives

ESD represents a promising technology, merging energy storage with smart window functionality. Although previous research has made some progress in practical applications, several challenges impede their widespread adoption. The following are suggestions for future study directions in this area of research.

Firstly, we suggest the optimization of electrochromic performance without compromising the supercapacitor’s energy storage capability. The dual functionality of ESD demands a delicate balance between transparency modulation and energy storage efficiency. Achieving this balance requires innovative electrode designs and precise control of material properties. Advanced fabrication techniques such as layer-by-layer assembly and nanostructuring could offer potential solutions by enabling precise control over the material architecture.

Secondly, we suggest the development of flexible and wearable ESD. Flexible supercapacitors with electrochromic functionality could be integrated into smart textiles and portable electronics, opening new avenues for energy storage and display technologies. Research in this domain involves the design of flexible substrates, stretchable electrodes, and robust encapsulation methods to ensure device performance under mechanical deformation.

Thirdly, we suggest the construction of large-size smart windows with energy storage functions. For an ESD in the application of large-size energy storage smart windows, the device needs to have a higher energy storage capacity and a wide voltage window. This requires the development of preparation methods that can precisely control the nano-morphology and size of the material to prepare electrode materials with high energy storage properties. At the same time, in order to improve the voltage window range of the device, it is also necessary to develop new electrolyte materials to meet the needs of high voltage range and high energy density.

Overall, ESD applications in the field of multifunctional energy storage devices and smart wearables are an interesting and rapidly evolving area of research. With the joint efforts of all parties, it is believed that many electrochromic energy storage materials can be realized in ESD practical applications, which is of great significance for the development of new energy storage devices in the future.

## 8. Conclusions

From the above discussions, it is evident that the integration of electrochromic functionality with supercapacitor technology offers a dual-purpose system that not only stores energy efficiently but also provides an immediate visual indication of the state of charge. The electrode materials used for ESD construction mainly include inorganic materials and organic materials. Organic materials have rich color palettes, rapid response, and ease of manufacture by solution treatment, however, they exhibit poor chemical stability compared to inorganic materials, thus limiting their practical application. Inorganic materials have long-term durability, a wide operating temperature range, and good chemical stability. However, due to the low electrical conductivity, high volume expansion during cycling, and low ion transport efficiency of inorganic materials, the inorganic ESD electrode materials have the disadvantages of slow color response and low energy storage capacity. Additionally, the prepared organic/inorganic nanocomposites can combine the advantages of organic materials and inorganic materials, so that the composite exhibits better energy storage performance, electrochromic performance, and cycle stability than a single material. It is believed that with the deepening of research, many electrochromic energy storage materials can be realized in the practical application of ESD

## Data Availability

Data is contained within the article.
